# Meaning in life and perceived quality of life in Switzerland: results of a representative survey in the German, French and Italian regions

**DOI:** 10.1186/s12955-015-0353-y

**Published:** 2015-09-29

**Authors:** Mathieu Bernard, Giliane Braunschweig, Martin Johannes Fegg, Gian Domenico Borasio

**Affiliations:** Palliative Care Service, CHUV, University of Lausanne, Lausanne, Switzerland; Department of Palliative Medicine, Ludwig-Maximilians University, Munich, Germany

**Keywords:** Meaning in life, General population, Quality of life

## Abstract

**Background:**

The concept of meaning in life (MIL) has become a central one in recent years, particularly in psycho-oncology and palliative care. The Schedule for Meaning in Life Evaluation (SMILE) has been developed to allow individuals to choose the life areas that they consider to be important for their own MIL. This approach relates to the “World Health Organisation” definition of quality of life (QOL) as an individual’s perception of his own position. The aims of this study were (i) to assess MIL in a representative sample of the Swiss population according to the three linguistic regions and (ii) to evaluate whether MIL constitutes a significant determinant of the perceived QOL.

**Methods:**

A telephone survey of the Swiss population, performed by a professional survey company, was conducted between November and December 2013. The interview included the SMILE, perceived QOL (0–10) and health status (1–5), and various sociodemographic variables. In the SMILE, an index of weighting (IOW, 20–100), an index of satisfaction (IOS, 0–100), and a total SMILE index (IOWS, 0–100) are calculated from the areas mentioned by the participants as providing MIL.

**Results:**

Among the 6671 telephonic contacts realized, 1015 (15 %) participants completed the survey: 405 French, 400 German and 210 Italian participants. “Family” (80.2 %), “occupation/work” (51 %), and “social relations” (43.3 %) were the most cited MIL-relevant categories. Italian participants listed “health” more frequently than German and French participants (50.4 % vs 31.5 % and 24.8 % respectively, χ^2^ = 12.229, *p* = .002). Age, gender, education, employment, and marital status significantly influenced either the MIL scores or the MIL-relevant categories. Linear regression analyses indicate that 24.3 % of the QOL variance (*p* = .000) is explained by health status (B = .609, IC = .490-.728, *p* = .000), MIL (B = .034, IC = .028-.041, *p* = .000) and socioeconomic status (F = 11.01, *p* = .000).

**Conclusion:**

The major finding of our analysis highlights the positive and significant influence of MIL on the perceived QOL in a representative sample of a general, multilingual and multicultural population. This result indicates that the existential dimension is not only determinant for QOL in some critical life events, as shown e.g. in psycho-oncology and palliative care, but also in everyday life.

## Background

The concept of meaning in life (MIL) has become a central one in recent years, particularly in psycho-oncology and palliative care and results showed that MIL contributes to the patients’ quality of life (QOL) [1–7]. In the clinical psychiatric perspective, the construct of MIL was first introduced by the Austrian psychiatrist Viktor Frankl, who defined MIL as the manifestation of values which are based on (i) creativity, (ii) experience (e.g. nature, love), and (iii) attitude (one’s attitude toward suffering and existential problems). Individuals are naturally and strongly disposed to search and find meaning and to feel that their life is therefore worthwhile [8]. In addition to Frankl’s definition, two other main explanations of MIL can be found: first, Reker and Wong [9] considered the cognitive, affective, and behavioural aspects of meaning and defined MIL as a “multidimensional construct consisting of the cognisance of order, coherence, and purpose in one’s existence, the pursuit and attainment of worthwhile goals, and the accompanying sense of fulfilment”. Secondly, Baumeister [10] proposed a definition of MIL as “a mental representation of possible relationships among things, events, and relationships”. The latter description was largely taken over in the “meaning making” model developed in the context of adjustment to stressful events such as incurable diseases [11, 12].

A recent review provided an overview of existing quantitative measures explicitly referring to meaning [13]. The instruments were categorized in two subgroups: a quantitative/deductive approach (nomothetic measurement, based on preselected dimensions) versus. a qualitative/inductive approach (idiographic approach, where free answers are first generated from the respondents before being classified and rated in order to allow for quantitative comparisons). Among the few instruments using the idiographic approach, the Schedule for Meaning in Life Evaluation (SMILE) has been developed to allow individuals to choose the life areas that they consider to be important for their own MIL. This approach relates to the World Health Organisation definition of QOL as an individual’s perception of the position in life in the context of the culture and value system where people live, and in relation to their goals, expectations, standards and concerns [14]. In this way, this QOL construct includes all the significant areas of life that allow people to achieve their goals and satisfy their needs at these different levels.

Based on the SMILE questionnaire, MIL was evaluated in a representative sample of the German population [15]. The results highlighted a difference between the relevant areas contributing to MIL according to age, with e.g. social relationships representing the most important areas in youth and young adult populations and values, spirituality and nature being the most mentioned areas in advanced age. No data could be found in the literature referring to the sources of meaning in the Swiss population, where on a small territory a representation of three major European linguistic and cultural backgrounds can be found (65 % German speaking, 23 % French, 8 % Italian, 9 % other).

The objectives of this study were:

(i) to assess MIL in a representative sample of the Swiss population, taking into consideration the possible differences between the three linguistic regions and the impact of several sociodemographic parameters.
(ii) to evaluate whether, in the general Swiss population, MIL represents a significant determinant of the perceived QOL, taking into account sociodemographic parameters and general health status.

## Methods

This study is one part of a broader palliative care research project, which aims to compare, inter alia, MIL in palliative patients and in a representative sample of the Swiss population. A telephone survey of the Swiss population with the SMiLE, performed by a professional survey company, was conducted between November and December 2013, resulting in a representative sample across the three linguistic regions. All interviewers of the professional survey were trained by two study investigators and received a standardized protocol including an assessment of MIL, QOL, health status and sociodemographic data: gender, age, education, employment, marital status, profession, self evaluation of the socioeconomic status, residence (according to the population size) and linguistic region. The following sampling technique was used: Firstly, households were drawn. Sampling at this stage was stratified by linguistic region and town size. The selected households were called and, when reached, the interviewer tried to establish its composition. The selection of the respondent among the household members (if household size >1) was then performed by quota (sex-age interlocked). All participants were informed that this survey was run on behalf of the palliative care Service of the University hospital of Lausanne and concerned perceived MIL, QOL and general health status. It was also specified that the data would be anonymised and that the results may be published in scientific journals. Participants were then interviewed only with their verbal consent. Individuals aged 16 years and older were eligible for the study.

### The Schedule for Meaning in Life Evaluation (SMILE)

Respondents are first asked to indicate from three to seven areas (*n *= number of areas) that actually provide meaning to their lives.

In a second step (level of importance), the importance of each area (w_1_…w_n_; 3 ≤ n ≤ 7) is rated with a five-point adjectival scale, ranging from 1 “somewhat important” to 5 “extremely important”.

In a third step (level of satisfaction), the respondents rate their current level of satisfaction with each area (s_1_…s_n_; with 3 ≤ n ≤ 7) on a seven-point Likert scale, ranging from −3 “very unsatisfied” to +3 “very satisfied”.

The Index of Weighting (IOW) indicates the mean weighting of the MiL areas (range, 20–100, with higher scores reflecting higher weights). Since the scale starts with “somewhat important”, the floor is set to 20 instead of 0.

$$ IoW=20\circ \frac{wges}{n};\kern0.5em {\mathrm{w}}_{\mathrm{ges}}={\displaystyle \sum_{i=1}^n{w}_i}. $$

The Index of Satisfaction (IOS) indicates the mean satisfaction or dissatisfaction with the individual MiL areas (range, 0–100, with higher scores reflecting higher satisfaction). To obtain a clear index varying from 0 to 100, the satisfaction ratings s_i_ are recalculated (s’_i_). “Very unsatisfied” (s_i_ = −3) is set to s’_i_ = 0 and “very satisfied” (s_i_ = +3) is set to s’_i_ = 100 with the levels of 16.7, 33.3, 50, 66.7, and 83.3 in between.

$$ IoS=\frac{{\displaystyle \sum_{i=1}^ns{\hbox{'}}_i}}{n}. $$

In the total SMiLE index (Index of Weighted Satisfaction, IoWS), the ratings for importance and satisfaction are combined (range, 0–100, with higher scores reflecting higher MiL).

$$ IoWS={\displaystyle \sum_{i=1}^n\left(\frac{w_i}{w_{ges}}\circ s{\hbox{'}}_i\right)}. $$

Levels and weights assigned to particular areas are theoretically independent and can change independently. A person may be satisfied in a particular area but assign little importance to it, while another area may be described at a high level of both importance and satisfaction. The psychometric proprieties of the SMILE in different countries (Germany, Spain and India) have been reported [3, 15–18]. This instrument was largely used, in particular in the health area, during the recent years [19–22].

The relevant areas contributing to MIL were classified in one of 15 categories^1^ based on this German nationwide survey and reported in a specific manual (http://www.psychotherapie-muenchen.de/downloads/SMiLE_Manual.pdf) developed for this instrument:

### Perceived QOL and perceived health status

After completion of the SMILE questionnaire, participants were asked to rate their perceived QOL and their perceived health status on numeric rating scales (range 1–10 (lowest possible QOL - highest possible QOL) for QOL and range 1–5 (very poor health – very good health) for health status). The validity of this approach has been shown previously [23–26].

### Statistical analysis

Comparisons between the three linguistic regions regarding the IOW, the IOS, and the IOWS were performed using independent sample T-tests or One-way ANOVA tests. Chi-square tests were used to identify potential differences between the regions in the likelihood of listing a specific MIL area. In order to be consistent with the representativeness in the total Swiss population, a weighted score was provided by the survey company to each participant taking into account his/her region, age, and gender. Indeed, women, French and Italian speaking regions, and respondents aged 60 years or more have been overrepresented in the surveyed sample in order to get a sufficiently large database. All weighting variables where calibrated on official demographics and interlocked (=weighting matrix of 2 × 9 × 3 = 54 cells). Data were then stratified according to the three regions, and a second weighted score was provided to each participant based on age and gender. Non-parametric tests (Mann–Whitney or Kruskal-Wallis), in case of categorical variables, or linear regression model, in case of continuous variables, were performed to assess potential differences between the sociodemographic variables regarding the three SMILE scores (IOS, IOW and IOWS). Chi-square tests were used to identify potential differences in the likelihood of listing a specific MIL area. All p-values were Bonferroni corrected (*p* ≤ .016 for the SMILE scores and *p* ≤ .003 for the MIL categories).

Considering our second objective (MIL as a potential determinant of QOL), linear regressions using the method of least squares were used. In a first step, and because MIL represents our variable of interest, a univariate regression analyse was performed with QOL as the dependent variable and MIL as the independent variable. In a second step, linear regressions were successively performed by adding to MIL each sociodemographic variable (gender, age, linguistic region, marital status, education, perceived socioeconomic status, residence, employment) and health status one by one. The variables with *p* < .05 or the variables influencing the MIL’s B coefficient (at least 10 % change) were selected in prevision of a complete multivariate model. In a third step, the complete model was tested, first by considering all the variables selected in step two and secondly by excluding the variables with *p* >.05. The most parsimonious model was finally chosen by considering also the adjusted R^2^. Age and health status were considered as continuous variables. Because gender, linguistic regions, marital status, education, socioeconomic status, residence, and employment include different modalities, dummy variables were created with one modality used as the reference.

Differences were considered to be statistically significant at *p* < .05. All *p*-values were Bonferroni corrected. Statistical tests were performed with the Statistical Package for Social Sciences (SPSS), version 22.

## Results

### Participation in the representative survey and respondents’ characteristics

For this representative survey of the Swiss population, 6671 telephonic contacts were realized: 1494 (22 %) individuals refused to participate, 2819 (42 %) individuals were unreachable, 488 (7 %) were out of “quotas”, 855 (13 %) presented various problems (language, invalid phone numbers, etc.), 2 were interviewed but did not fully complete the questionnaire and finally 1015 (15 %) individuals completed all questionnaires (405 French participants, 400 German participants and 210 Italian participants).

Taking into account the representativeness of the total Swiss population according to the region, age and gender, the repartition between the 3 linguistic regions was *n* = 715 (70.5 %) for the German part, *n* = 256 (25.2 %) for the French part and *n* = 44 (4.3 %) for the Italian part. Table 1 provides an overview of the respondents’ characteristics according to the weighted score.

**Table 1:** Respondents’ characteristics

	Total / *n* = 1014		German / *n* = 715		French / *n* = 255		Italian / *n* = 44	
	n	%	n	%	n	%	n	%
Age								
16–19 years	54	5.3	37	5.1	15	5.8	2	4.7
20–29 years	155	15.3	109	15.2	41	15.9	6	12.6
30–39 years	166	16.4	117	16.4	43	16.8	7	14.9
40–49 years	193	19.0	135	18.9	49	19.1	9	19.8
50–59 years	167	16.5	119	16.7	41	16.0	7	16.2
60–69 years	132	13.0	94	13.1	32	12.5	6	14.2
70–79 years	88	8.7	63	8.8	21	8.2	5	10.8
80–89 years	49	4.8	35	4.9	12	4.6	2	5.6
90–99 years	10	1.0	7	0.9	2	1.0	1	1.3
Gender								
Male	501	49.4	353	49.4	126	49.5	22	49.4
Female	513	50.6	362	50.6	126	50.5	22	50.6
Marital status								
Single	270	26.7	187	26.2	73	28.9	10	22.2
Married/Legal Partnership	600	59.2	432	60.3	141	55.7	27	62.7
Divorced/Separated	68	6.8	39	5.5	26	10.3	3	7.1
Widowed	73	7.2	57	7.9	13	5.1	3	7.5
Missing	2	0.2			2	0.9		
Education								
Primary , secondary school (elementary)	144	14.2	95	13.3	42	16.6	7	15.7
Professional school/ Apprenticeship/High school (secondary)	532	52.5	386	54.0	120	47.1	26	60.0
Vocational school / University (high)	326	32.2	224	31.4	31	35.9	11	24.1
Missing	11	1.0	10	1.4	1	0.4		
Occupational status								
Full time	408	40.3	294	41.0	100	39.1	15	34.6
Part time	252	24.9	185	25.8	58	22.9	9	21.0
No professional activity	353	34.8	237	33.1	97	38.0	19	44.4
Socioeconomic status								
Lower class	181	17.9	118	16.5	51	20.7	12	28.0
Lower middle class	191	18.8	129	18.1	53	46.5	9	19.9
Upper middle class	547	53.9	410	57.3	118	10.8	19	43.0
Upper class	74	7.3	44	6.2	27	2.1	3	6.8
Missing	20	2.0	14	2.0	5	2.1	1	2.4
Residence (population size)								
>100’000	112	11.0	77	10.8	35	13.8	0	0
50’000–99’999	42	4.1	34	4.8	32	12.5	8	17.1
20’000–49’999	98	9.6	66	9.2	85	33.3	0	0
5000–19’999	342	33.8	247	34.5	0	0	11	24.6
<1000–4999	420	41.4	292	40.8	103	40.4	26	58.3

### Differences in MIL according to the three linguistic regions

#### Total sample

##### IOW, IOS and IOWS SMILE scores

After Bonferroni correction (*p* < .016), one-way Anova tests indicated no significant differences between the three regions for the SMILE scores (see Table 2 for an overview of the mean scores according to the three regions).

**Table 2:** SMILE scores regarding the regions (*N* = 1014)

	Total	German	French	Italian	F	P
		M ± SD	M ± SD	M ± SD		
IOW score (range: 20–100)	82.9 ± 11.3	83.3 ± 10.9	81.4 ± 12.2	84.6 ± 11.8	3.333	.036
IOS score (range: 00–100)	85.9 ± 13.1	86.2 ± 13.2	85.2 ± 12.8	84.6 ± 13.4	.770	.463
IOWS score (range: 00–100)	86.5 ± 13.6	86.7 ± 14.0	86.2 ± 12.7	85.7 ± 13.1	.191	.826

*IOWS* Index of Weighted Satisfaction (total SMILE score), *IOW* Index of Weighting (importance score), *IOS* Index of Satisfaction (satisfaction score)

##### MIL categories

In total, 4164 areas were listed by the 1015 respondents. All the answers were translated into English before two independent raters (GB and MB) assigned separately all the mentioned areas to one of the 15 categories described above. Where there was disagreement between the two raters, discussion was undertaken until a consensus was reached. A category labelled as “other” was added for the areas that could not be included in one of the 15 predetermined categories.

Table 3 shows frequencies and percentages of the MIL areas listed by the respondents in total and separately in the three regions. After Bonferroni correction (*p* ≤ .003), Chi-square tests indicate significant differences for “health”: Italian participants listed “health” more frequently than German and French participants (50.4 % vs 31.5 % and 24.8 % respectively). Tendencies were found for “spirituality” (German speaking > French and Italian), nature/animals (German > French > Italian) and “satisfaction” (Italian > German and French).

**Table 3:** Areas of MIL listed by the respondents including number and percentage of the respondents (*N* = 1014)

	Total		German		French		Italian		χ^2^	p
	n	%	n	%	n	%	n	%		
Family	813	80.2	579	80.9	199	78.3	35	79.9	.760	.684
Partnership	164	16.2	112	15.6	43	16.8	10	22.3	1.620	.445
Social relations	439	43.3	315	44.1	109	42.6	15	35.3	1.438	.487
Occupation/work	517	51.0	366	51.2	133	52.3	18	40.8	1.991	.370
Leisure time	423	41.7	296	41.4	116	45.4	12	26.6	5.291	.071
Home/garden	66	6.6	51	7.2	14	5.6	1	2.2	2.168	.338
Finances	102	10.1	78	10.8	18	7.1	7	15.3	4.707	.095
Spirituality/religion	131	12.9	107	15.0	21	8.2	3	6.0	9.089	.011
Health	311	30.6	225	**31.5**	63	**24.8**	22	**50.4**	12.229	**.002**
Satisfaction	85	8.4	55	7.6	21	8.4	9	21.0	8.812	.012
Nature/animals	226	22.3	178	24.8	44	17.3	5	10.7	9.470	.009
Social commitment	35	3.5	26	3.7	7	2.7	2	4.5	.613	.736
Hedonism	78	7.7	48	6.8	26	10.3	3	7.2	3.289	.193
Art/culture	160	15.8	101	14.2	53	20.9	6	13.4	6.563	.038
Growth	73	7.2	47	6.6	23	9.0	3	6.2	1.694	.429
Other	146	14.5	97	13.5	24	9.2	11	25.5	7.592	.024

Bold data: Significance test when *p* ≤ .003

#### German speaking region^2^

Regarding the SMILE scores, significant differences were found for gender (women higher than men), age (older higher than younger), education (high education level lower than low education level), marital status (single participants lower than other participants).

Regarding the listed categories, significant differences were observed for gender (women mentioned more “family” than men), age (younger mentioned more “social relations” than older and middle-aged mentioned more “work occupation” than younger and older), marital status (single participants mentioned more “social relations” than other participants, and separated participants mentioned more “health” than other participants), and employment (unemployed participants mentioned “health” and “satisfaction” more than employed participants).

#### French region

Regarding the SMILE scores, significant results were found for gender (women higher than men) and socioeconomic status (“lower classes” lower than “higher classes”).

Regarding the listed areas, significant differences were observed for education (high education level mentioned more “art/culture” than low education level), employment (employed participants mentioned “family” and “work/occupation” more than unemployed participants), marital status (married participants mentioned more “family” than other participants, single participants mentioned more “partnership” and “hedonism” than other participants, and married/widowed participants mentioned more “health” than other participants), socioeconomic status (middle class mentioned more “family” than lower and higher class) and age (middle-aged participants mentioned more “family” than other participants, and older participants mentioned more “nature”, “health” and “work/occupation” than other participants).

#### Italian region

Regarding the listed areas, significant results were found for employment (employed participants mentioned more “work/occupation” than unemployed participants), marital status (widowed participants mentioned less “occupation/work” than other participants, and age (older participants mentioned more “occupation/work” than other participants).

Table 4 shows a summary table of the sociodemographic variables impacting MIL according to the three linguistic regions.

**Table 4:** Overview of the sociodemographic variables impacting the SMILE scores and MIL categories

	German	French	Italian
SMILE scores	Gender	Gender	
	Age	Socioeconomic status	
	Education		
	Marital status		
MIL categories	Gender	Age	Age
	Age	Education	Employment
	Employment	Employment	Marital status
	Marital status	Marital status	
		Socioeconomic status	

SMILE scores include IOWS (Index of Weighted Satisfaction), IOW (Index of Weighting score), and IOS (Index of Satisfaction score). SMILE categories include family, partnership, social relations, occupation/work, leisure, home/garden, finances, spirituality/religion, health, satisfaction, nature/animals, social commitment, hedonism, art/culture, growth, and other

### MIL as a predictor of QOL

In a first step, a univariate regression analyse was performed with QOL as the dependent variable and MIL as the independent variable. The result of the linear regression showed that the model explained 12.1 % of the variance of the perceived QOL (adjusted R^2^ = .121, *p* = .000) and that MIL appeared as a significative predictor of QOL (see Table 5).

**Table 5:** Linear regression between perceived QOL and MIL

Parameters	Coefficients B	95 % Confidence interval for B		Std. error	T value	p
Constant	3.777	3.141	4.413	.324	11. 65	.000
IOWS	.043	.036	.051	.063	11.80	.000

*IOWS* Index of Weighted Satisfaction (total SMILE score)

In a second step, in order to evaluate which variable will be included in the multivariate model, linear regressions were successively performed by adding to MIL each sociodemographic variable (gender, age, linguistic region, marital status, education, perceived socioeconomic status, residence, employment) and health status one by one. The results indicated that only the health status (adjusted R^2^ = .217, *p* = .000) and the perceived socioeconomic status (adjusted R^2^ = .164, *p* = .000) appeared as influencing significantly the relationship between QOL and MIL (see Tables 6 and 7 below).

**Table 6:** Linear regression between perceived QOL and (i) MIL and (ii) health status

Parameters	Coefficients B	95 % Confidence interval for B		Std. error	T value	p
Constant	1.817	1.124	2.510	.353	5.14	.000
IOWS	.034	.028	.042	.003	9.74	.000
Health status	.679	.560	.798	.060	11.23	.000

*IOWS* Index of Weighted Satisfaction (total SMILE score)

**Table 7:** Linear regression between perceived QOL and (i) MIL and (ii) socioeconomic status

Parameters	Coefficients B	95 % Confidence interval for B		Std. error	T value	p
Constant	3.463	2.818	4.107	.328	10. 54	.000
IOWS	.042	.035	.049	.004	11.62	.000
Socioeconomic status						
Lower middle class	.096	−.201	.394	.152	0.64	.525
Upper middle class	.696	.450	.942	.125	5.56	.000
Upper class	.888	.490	1.286	.203	4.38	.000

*IOWS* Index of Weighted Satisfaction (total SMILE score)

In a third step, the complete model including MIL, health status and socioeconomic status was tested. The result of the linear regression showed that the complete model explained 24.3 % of the variance of the perceived QOL (adjusted R^2^ = .243, *p* = .000) and that MIL, health status and socioeconomic status appeared as a significative predictors of QOL (see Table 8).

**Table 8:** Linear regression between perceived QOL and (i) MIL, (ii) socioeconomic status, and (iii) health status

Parameters	Coefficients B	95 % Confidence interval for B		Std. error	T value	p
Constant	1.782	1.083	2.482	.356	5. 00	.000
IOWS	.034	.028	.041	.004	9.76	.000
Health status	.609	.490	.728	.061	10.04	.000
Socioeconomic status						
Lower middle class	.042	−.242	.327	.144	0.29	.771
Upper middle class	.532	.296	.769	.120	4.42	.000
Upper class	.730	.349	1.110	.194	3.76	.000

*IOWS* Index of Weighted Satisfaction (total SMILE score)

Because a difference between lower class and upper middle class (higher QOL in the upper middle class, *p* = .000) and a difference between lower class and upper class (higher QOL in the upper class, *p* = .000) were observed, a total test was computed for the socioeconomic status in order to assess if QOL changes according to the four classes. The total region test indicated a significant impact of the socioeconomic status on QOL (F = 11.01, *p* = .000): regression tests indicated a significant difference between the lower middle class and the upper middle class (higher QOL in the upper middle class, B = .490, *p* = .000) and between lower middle class and upper class (higher QOL in the upper middle class, B = .687, *p* = .000).

## Discussion

After the study of Fegg et al. [15], this is the second nationwide survey on MIL in a representative general population and the first, to our knowledge, to assess the impact of MIL on perceived QOL in a general population.

### MIL in the Swiss population

Concerning our first aim, our results showed high IOW, IOS and IOWS scores for the total population and for all the three linguistic regions, reflecting a globally high satisfaction with their MIL. This result corresponds to the results of Fegg et al. [15] with the German population. Similarly to the German study, the four most mentioned categories in the total Swiss population are “family”, “occupation/work”, “social relations”, and “leisure time”, while the least cited categories were “satisfaction”, “hedonism”, “growth”, and “social commitment”.

To our knowledge, only few studies have evaluated potential differences in the relevance of existential domains between these three main Swiss linguistic regions. Switzerland took part in the “World Value Survey” in 1988–1989 [27]. The survey also evaluated the priority areas in life in terms of importance and significance, similar to the SMILE approach. The results showed that “family” was the most important area in the three linguistic regions of Switzerland (95 % of the respondents), followed by “friends” (90 %), “professional activity” (87 %), “leisure” (84 %), “religion” (55 %) and “politics” (39 %). Only minor differences between the three regions were observed. A noteworthy difference between our results and those of the values survey concerns the religion/spirituality area. This category was only mentioned by a minority of our participants in all three regions (12 % on average in our total sample). A possible explanation of this discrepancy could be the development of secularism in the last 25 years in Switzerland, as well as in the majority of European countries [28].

When considering the predominant categories observed in our study (“family”, “occupation/work”, “social relations” and “leisure time”), the dominance of the individualistic values appears, which refers to personal issues depending on individual responsibility and development that do not aim to impact primarily on the social interest or the common good. According to the major theories in cross-cultural research, these life areas represent the shift between societies characterized by religious and survival oriented attitudes and values, and secular societies essentially concerned by the development and valorization of the individual well-being [29]. With reference to the theory of basic human values developed by the social psychologist Shalom Schwartz, a parallel can also be established between these individualistic areas centered on personal needs and the so-called “openness to change” dimension, which integrates the “hedonism”, “stimulation” and “self-direction” values and appears to be the most prevalent dimension in Switzerland, based on the results of the European social survey [30, 31]. The “self-direction” values in particular seem to reflect the individualistic values mentioned above, since they are defined as a response to the basic needs of autonomy and independence associated with the individual development process. By referring to the self-determination theory (SDT), a general theory of human motivation applied in many different domains (health, education, work, sport), autonomy contributes to individual wellbeing by facilitating more independent forms of behavioral regulation [32, 33].

Our results indicate only few differences between the three linguistic regions when considering the areas contributing to MIL: only the “health” category was clearly over-represented in the Italian-speaking region. Based on the value profile of 20 European countries [34], those with common cultural and historical backgrounds tend to share the same values. This finding may explain, at least in part, why the differences observed between the three linguistic regions of Switzerland are relatively small. Regarding the influence of sociodemographic variables on MIL, results are relatively similar in the three regions: education, employment, marital status and age have an impact on either the MIL scores or the mentioned areas contributing to MIL in the three regions. Age and education were also found in the study of Fegg et al. [15]. The differences observed in the categories mentioned according to age seem also correspond to the Erikson’s phases of the psychosocial development, with life-stage challenges that are specific to each age step: social relations categories are more often cited by young participants, work occupation by respondents between 30 and 50 and health by older participants [35]. Gender was also identified as an influential variable, with women reporting a higher MIL score, but the difference was statistically significant only in the French sample. Another difference with the results of Fegg et al. [15] is the absence of the influence of the residence. Whereas in Germany participants were more satisfied in rural areas and small cities, our results do not indicate such a tendency in Switzerland. A possible explanation lies in the fact that the contrast between villages, small cities and big cities is less pronounced in Switzerland than in Germany.

### MIL as a predictor of QOL

Not surprisingly, the perceived health status represents a major predictor of the perceived QOL in the Swiss population. In the literature concerning general populations, the areas of life considered as important for QOL are: social relationships, activities and participation, physical, environment, and psychological areas [2, 36, 37]. Among these, the physical domain (e.g., health status, absence of chronic disease or multimorbidity), represents one of the most important determinants of QOL [38–40]. In an international investigation focusing on older adults, many of the physical aspects were also measured as the most determinants factors for QOL [36].

The major finding of our analysis highlights the positive and significant influence of MIL on the perceived QOL. To our knowledge, there are no studies conducted with general population that address the relationship between QOL and MIL, and more specifically how MIL may contribute to QOL. So far, the link between existential and QOL has been investigated predominantly in the medical context, e.g. in psycho-oncology and palliative care. Cohen et al. [7] highlighted that the existential wellbeing was at least as important as any other domain in predicting the overall perceived QOL for palliative patients. Numerous studies have shown that QOL at the end of life is closely linked to non-physical determinants [3, 6, 41–43]. In reviews focusing on the QOL domains that are important for incurably ill patients, Albers et al. showed that spiritual/existential well-being was one of the most often cited domains [1, 44]. Many studies have already shown that MIL may represent an efficient protection factor against the development of depression, anxiety, psychological distress, and desire for hastened death when facing a life threatening illness [4, 5, 45, 46]. Our results support the notion that MIL is a preeminent factor influencing QOL in the general population.

Among the other sociodemographic variables, only the perceived socioeconomic status showed a significant positive correlation with QOL. This result is in agreement with other recent studies [38, 47]. It is worth mentioning that the total variance explained by our model is lower than the variance explained in other studies specially focused on the identification of the significant factors of QOL: 24 % in our study versus 40–45 % [36, 38, 48]. Compared with these studies, this difference may probably be explained in a large part by the non-inclusion of specific predictors that have been shown to be particularly relevant for QOL, e.g. personality trait and social support.

### Conclusion and limitations

This study has several limitations. First, as reported by Fegg et al. [15], it is possible that the interview strategy (telephone interviews) may account for some divergences in the participants’ responses. Face to face interviews would have allowed a greater depth in the eliciting of the personal areas that contribute to personal MIL. The great advantages of a telephone-based survey are the cost-effectiveness and the possibility of comparing the two studies. Secondly, the categories associated with the areas cited by the respondents were assigned “a posteriori”, which retains a degree of subjectivity by the investigators. Thirdly, it was a deliberate choice to address the concept of MIL with an essentially subjective and non-theoretically driven methodology. Since there is no consensus with regard to the definition of MIL, we chose to stay close to the definition suggested by Fegg et al. [15] and paraphrasing Ciaran O’Boyle’s definition of individual QOL: “meaning in life is whatever the individual says it is” [49]. It is therefore likely that the areas mentioned by participants as contributors of MIL partly overlap the areas that contribute to QOL. Further studies could assess if the responses given by the respondents would differentiate themselves by considering on one side the SMILE and on the other side the Schedule for the Evaluation of Individual QOL (SEIQoL [50]), based on the same methodology. Further research aiming to investigate the link between MIL and QOL could use an instrument covering another aspect of the MIL construct, for example the presence/absence of MIL perception or the level of perceived distress in MIL (see ref. [13] for an overview of all the instruments assessing the MIL concept). Fourth, if the use of a single-item scale for the assessment of QOL is valid and justified in the medical context (for example in order to be the less time consuming for the patients who may experience many symptoms related to their illness), such an evaluation does not allow an in-depth and complete assessment of QOL. This should be tested in future research. Fifth, we cannot exclude an order effect due to the order in which the questions were presented to the participants. Sixth, the criteria considered for the recruitment of the participants according to the linguistic regions (speaking fluently the language of the targeted region and living in it) did not allow for taking into account any cultural information and integration, which may also play a role in the MIL’s interpretation. Finally, the survey was conducted in a two-month period at the beginning of winter. This short period does not allow to consider a potential impact of the seasons on the subjective interpretation of MIL.

In conclusion, our results indicate a high level of satisfaction with MIL in Switzerland and a significant influence of sociodemographic variables such as education, employment, marital status and age. Importantly, our data show the importance of MIL as a determinant of perceived QOL in the general Swiss population, illustrating the importance of the existential domain not only in crisis situations (e.g. life-threatening illness), but also in everyday life.

## Abbreviations

MIL: Meaning in life
QOL: Quality of life
WHO: World Health Organisation
SMILE: The Schedule for Meaning in Life Evaluation
IOWS: Index of Weighted Satisfaction (total SMILE score)
IOW: Index of Weighting (importance score)
IOS: Index of Satisfaction (satisfaction score)

## Footnotes

1 1. Family 2. Partnership 3. Social relations 4. Occupation/Work 5. Leisure time/Relaxation 6. Home/garden 7. Finances 8. Spirituality/Religion 9. Health 10. Satisfaction 11. Nature/Animals 12. Social commitment 13. Hedonism 14. Art/Culture 15. Growth.

2 Detailed statistical results can be requested to the principal author of the article.
